# Tandem Occlusion to Thrombolysis in Cerebral Infarction (TICI) 3: A Case of Perioperative Tirofiban for Carotid Artery Stenting

**DOI:** 10.7759/cureus.106147

**Published:** 2026-03-30

**Authors:** Laura Polhemus, Aaron Barkhordar, Adam Awad

**Affiliations:** 1 Department of Neurology, Saint Louis University School of Medicine, St. Louis, USA

**Keywords:** carotid artery disease, carotid stent, endovascular mechanical thrombectomy, endovascular stenting, stroke, stroke systems of care, stroke treatment, tirofiban

## Abstract

Large vessel occlusions associated with ipsilateral high-grade extracranial internal carotid artery stenosis, also called tandem occlusions, remain under investigation for optimal management. The risk of in-stent thrombosis is heightened for those who undergo carotid stenting in conjunction with mechanical thrombectomy (MT). Tirofiban, an intravenous antiplatelet agent functioning by binding the GIIb/IIIa receptors, inhibits platelet aggregation and has been used in cases of tandem occlusions to reduce the risk of in-stent thrombosis. Unfortunately, the bleeding risk is elevated in patients who receive intraoperative antithrombotics during stenting procedures, and especially those who undergo reperfusion therapy with prior fibrinolytic therapy. We present a case of tirofiban used to reduce the risk of in-stent thrombosis during MT and stenting of a carotid terminus tandem occlusion in a 64-year-old man. We aim to compare our case with currently published data on tirofiban use during similar procedures, with regard to indications for treatment, hemorrhagic events, and functional outcomes.

## Introduction

Large vessel occlusions associated with ipsilateral high-grade extracranial internal carotid artery (ICA) stenosis, also called tandem occlusions, can lead to devastating strokes due to the clot burden. The challenging nature of treating tandem occlusions is attributable to the clinical decision and expertise of opening the proximal stenosis or occlusion. Emergent stenting of the lesion remains an option, and has been associated with improved functional outcomes [1]; however, the risk of in-stent thrombosis is high. Administering intraoperative antiplatelets in the hyperacute setting via an intravenous (IV) route has been studied as a way to reduce this risk, as well as the risks of embolic migration and reocclusion [2,3]. However, the use of IV antiplatelets must be weighed with their risk of hemorrhage, as some trials have shown an increased rate of fatal intracerebral hemorrhage (ICH) with their administration in these settings [4].

Tirofiban is an IV antiplatelet agent that binds to the GIIb/IIIa receptors on platelets and inhibits the aggregation cascade. Previously studied in percutaneous coronary interventions, the use of tirofiban in cases of neurological ischemia came into interest in the early 2010s. One of the earliest randomized controlled trials assessing the safety of IV tirofiban was the Safety of Tirofiban in acute Ischemic Stroke (SaTIS) trial, in which patients with National Institutes of Health Stroke Scale (NIHSS) 4-18 (moderate stroke) presenting between 3 and 22 hours after stroke onset were given either tirofiban or placebo if not treated with IV alteplase. Results showed favorable outcomes in mortality but not functional outcomes. Notably, there was no increased risk of ICH [5].

Since then, further investigation into the use of tirofiban within the scope of endovascular procedures has surfaced. Most investigations focus on outcomes when using tirofiban during thrombectomy cases in which endothelial dysfunction is at higher risk, such as incomplete revascularization or low thrombolysis in cerebral ischemia score (TICI) ≤2b [6-8], stenting or angioplasty [3,4,7-9], and multiple pass attempts [4].

The case described in this article adds to the growing literature of patients with higher risk demographics who demonstrate safe outcomes when treated for tandem occlusions. We review previous trials using periprocedural tirofiban and their relationship to our case in the discussion, and specifically aim to compare our case with studies that include patients who presented with high stroke severity scores, received preprocedural thrombolysis, and underwent extracranial stenting. We also compare the dosing and duration of tirofiban to previous studies.

## Case presentation

**Figure 1:**
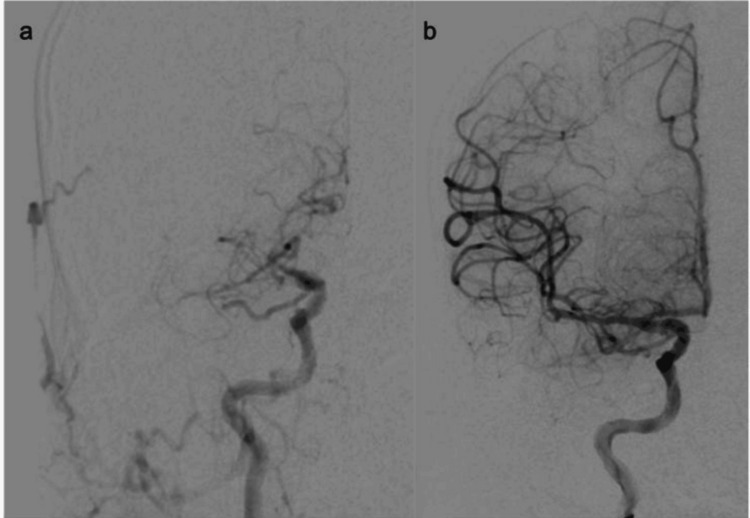
Digital subtraction angiography showing TICI 0 flow through the proximal right middle cerebral artery (a) and TICI 3 flow through the revascularized artery (b) TICI: thrombolysis in cerebral infarction

A 64-year-old man presented to an outside hospital in July 2024 with an acute onset of right gaze deviation and left hemiplegia after falling out of a chair at work. His initial NIHSS score was 8, and IV Tenecteplase was administered at an outside facility 70 minutes after symptom onset. He was transferred to our institution within an hour, and upon arrival, his NIHSS score had deteriorated to 20. A repeat head computed tomography (CT) scan revealed an Alberta Stroke Program Early Computer Tomography Score (ASPECTS) of 6 [10]. He was found to have a right middle cerebral artery (MCA), M1 segment occlusion on CT angiography, and later confirmed on digital subtraction angiography (Figure 1a). The patient was then taken for mechanical thrombectomy (MT) under general anesthesia.

**Figure 2:**
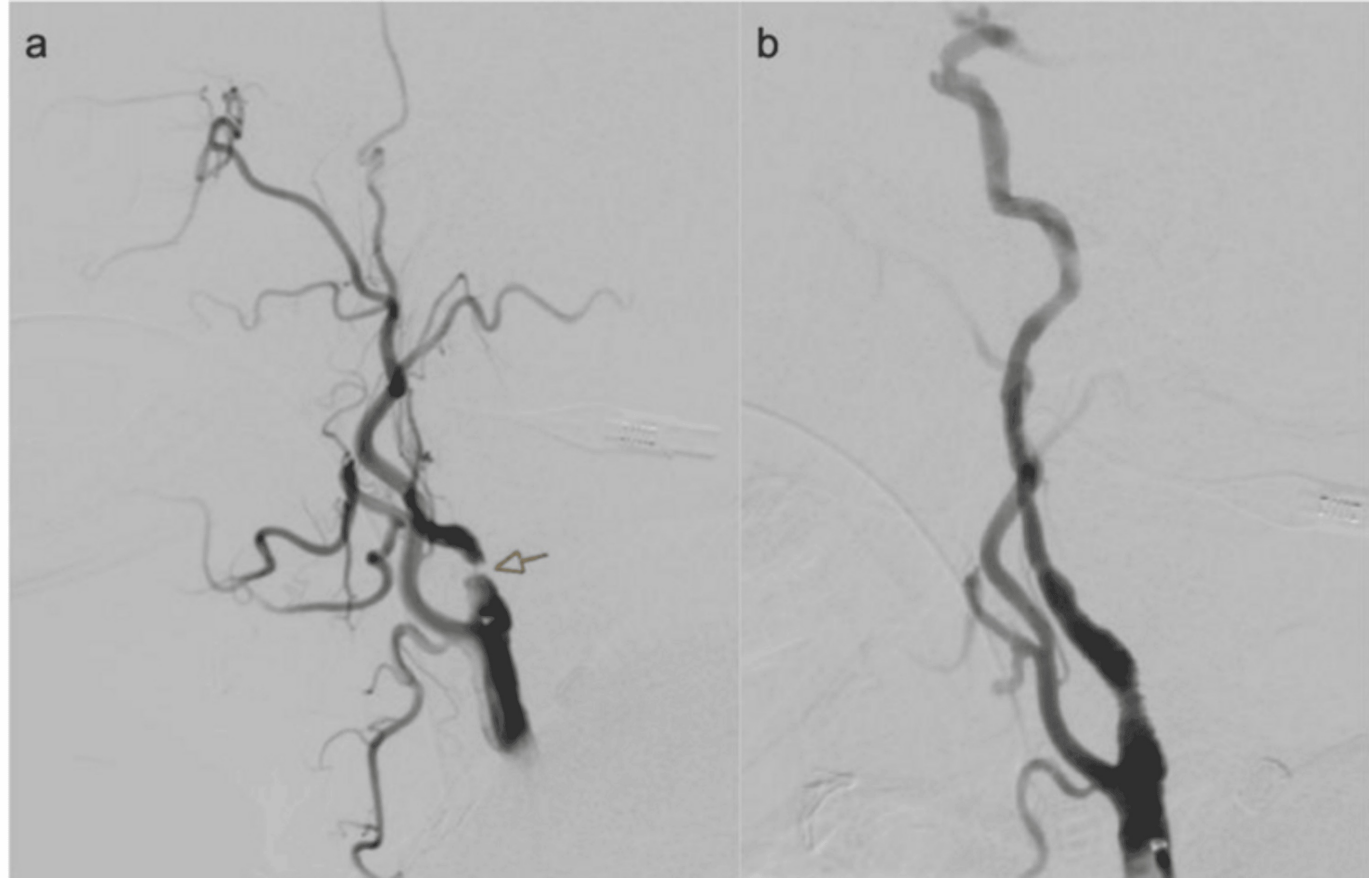
Digital subtraction angiography depicting severe stenosis of the right cervical internal carotid artery prior to revascularization (a; arrow) and postcarotid stenting (b)

Door-to-puncture was 36 minutes. During the procedure, the right cervical ICA was found to be severely stenotic (Figure 2a), and balloon angioplasty was performed. After the advancement of the system, the clot occluding the right M1 segment of the MCA was removed with an aspiration catheter via direct contact aspiration after the first pass, resulting in a thrombolysis in cerebral infarction (TICI) 3 outcome (Figure 1b). Door-to-reperfusion was 61 minutes (total time from symptom onset to recanalization was 199 minutes). The patient was given a 12 mcg/kg IV tirofiban bolus followed by a 0.1 mcg/kg/minute infusion (total body weight 100 kg), after which stenting of the right cervical ICA was performed (Figure 2b). The tirofiban infusion was continued for the duration of the procedure and 24 hours postoperatively.

**Figure 3:**
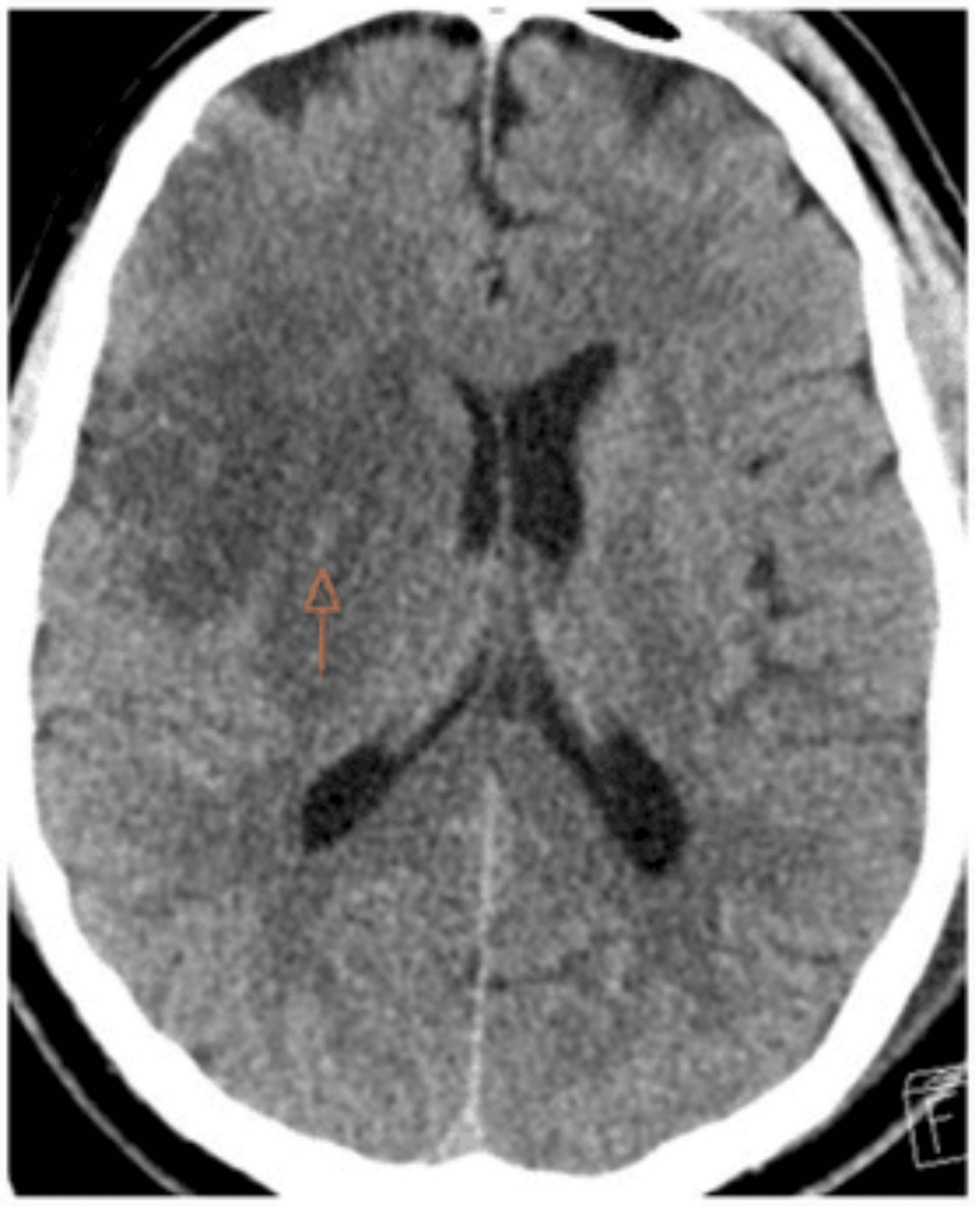
CT of the head without contrast 24-hour postinterventional treatment demonstrating hemorrhagic infarction type I (orange arrow) CT: computed tomography

**Figure 4:**
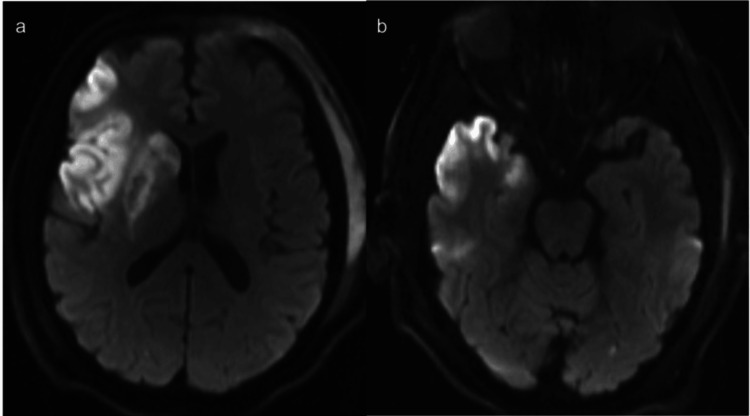
Diffusion-weighted MRI performed 24 hours after thrombectomy showing infarcts in the right basal ganglia and frontal lobe (a) and right temporal lobe (b)

The patient was successfully extubated the next day, and the NIHSS score improved to 8. A repeat CT scan 24 hours postoperatively displayed hemorrhagic infarction type 1 based on the Heidelberg classification system (Figure 3) [11], and MRI demonstrated a moderate-sized infarct in the right MCA territory (Figure 4). The patient was transitioned from tirofiban infusion to oral aspirin 81 mg and clopidogrel 75 mg daily after a 600 mg clopidogrel load, and started on high-intensity atorvastatin for secondary stroke prevention. He experienced unilateral headaches postoperatively, similar to chronic headaches that resolved with symptomatic therapy, and was transferred from the intensive care unit to the floor on hospital day 2. He was discharged to an acute rehabilitation facility on hospital day 5. His discharge NIHSS score was 0, and the modified Rankin scale (mRS) score was 4, with minimal assistance walking under supervision. At his two-month follow-up in the stroke clinic, the patient had returned to his baseline functionality with an mRS score of 2.

## Discussion

Our case demonstrates a patient who underwent MT and emergent stenting of a tandem occlusion using intraoperative IV tirofiban without symptomatic hemorrhage, a low-NIHSS score on discharge, and good functional recovery at 90 days. Several factors in our case are critical to compare with the published data, particularly preprocedural administration of a thrombolytic agent, high stroke severity score on presentation, and the dose and duration of tirofiban infusion. Large artery atherosclerotic etiology of stroke in our patient and procedural stenting are comparable to demographic data that have been published on patients who receive tirofiban during emergent therapy [3,4,6-9,12-14] and will be further discussed.

The patient in our case received thrombolytic therapy prior to being taken for endovascular treatment. The most notable data supporting this are the post hoc analysis of the DIRECT-MT trial, a landmark trial demonstrating the noninferiority of thrombectomy alone compared with thrombectomy with bridging therapy with IV alteplase [7]. The post hoc analysis looked at patients who had received tirofiban during thrombectomy and analyzed demographics, the interaction between recombinant tissue plasminogen activator, and outcomes. The authors found no statistically significant difference in primary or secondary functional outcomes, including 90-day mRS, nor ICH risk in the analysis of safety outcomes. Other subsequent trials that included patients who had received thrombolytic therapy prior to tirofiban treatment showed no significant increase in the rate of ICH [8].

Other factors that influenced clinical decision-making in our case and have been analyzed in previous articles were time to presentation and recanalization, presenting NIHSS score, and presenting ASPECTS score. A notable negative trial showing increased rates of fatal ICH published in 2013 included patients with high NIHSS scores of up to 35 (including posterior circulation strokes) and longer duration to recanalization [4]. Demographics of unequivocal trials included patients with similar baseline NIHSS but overall higher ASPECTS score [7,12] and longer duration from last known well to reperfusion [12]. The trial with baseline demographics most representative of our patients, including baseline NIHSS, reperfusion latency, and even addressing patients with tandem occlusion, was studied in patients with an even lower presenting ASPECTS score [8]. The results of this trial showed a significant improvement in 90-day mRS and NIHSS compared with patients treated with a placebo. These characteristics suggest that patients who present earlier and are treated more urgently with lower NIHSS scores on admission may have better functional outcomes, regardless of ischemic core.

The dose of tirofiban used in the hyperacute setting in our case was a 12 mcg/kg IV bolus followed by a 0.1 mcg/kg/minute infusion for 24 hours. In one of the earliest trials with tirofiban, the SaTIS trial, the dose and duration of IV tirofiban were 0.4 mcg/kg/minute for 30 minutes, followed by an infusion of 0.1 mcg/kg/minute for 48 hours [5]. There were no differences in the rates of cerebral hemorrhage between the tirofiban group and the placebo group. The SaTIS trial did not study patients who underwent MT or patients who received thrombolytic therapy; however, their pharmacological methods were carried forward and modified in the later endovascular trials. For example, the dose of IV tirofiban and subsequent bridging with dual antiplatelet therapy (DAPT) was almost identical between our case and the dose used in the Direct Intra-arterial thrombectomy in order to Revascularize AIS patients with large vessel occlusion Efficiently in Chinese Tertiary hospitals: a Multicenter randomized clinical Trial (DIRECT-MT), which was 0.1-0.4 mcg/kg/minute for 30 minutes or 12 mcg/kg IV bolus dose followed by 0.1 mcg/kg/minute for a 24-hour period with a six-hour overlap of DAPT [7]. Another retrospective case series of patients who underwent angioplasty with or without stenting following MT observed decreased rates of early reocclusion using only 12 hours of IV infusion at 0.1 mcg/kg/minute overlapping with DAPT and no increased risk of hemorrhage, suggesting that even without bolus and shorter duration of treatment that rates of reocclusion, which is the goal with administering intraoperative antiplatelet therapy, may be achievable. Other trials that studied intra-arterial administration of tirofiban showed decreased risk of death [6] and lower 90-day mRS score [8] and no increased risk of ICH compared with those not treated, and suggest this as a possible safe alternative as well.

More recent, specific studies on tandem occlusions are important for comparison with our patient’s case. Investigators looked at tandem lesions that underwent emergent stenting treated with tirofiban vs. tirofiban combined with another antithrombotic [12]. The dose of IV tirofiban used was a 25-mcg/kg bolus followed by 0.15-mcg/kg/minute infusion for 24 hours, which is considered a high dose and reflects cardiology practices in percutaneous coronary interventions and similarly used in the trial by Kellert et al. [4]. Most patients had similar baseline NIHSS scores to our patient, but a more favorable ASPECTS score on admission, and both groups received thrombolytic if eligible. Half of the patients treated with tirofiban only achieved an mRS of 0-2 at 90 days, and only one patient experienced symptomatic hemorrhage. In the entire patient cohort, factors that significantly influenced rates of symptomatic hemorrhage were higher NIHSS, tandem occlusions, and thrombolytic therapy. This data cannot be generalized to our patient due to the high dosage of tirofiban; however, factors that influence poor outcomes should be evaluated nonetheless when taking patients for intervention.

A newer study that is highly applicable to our clinical case is a retrospective analysis of the A Registry for Thrombectomy In Stroke Therapy from Andalusia registry, looking at patients who had atherosclerotic ICA stenosis treated with emergent carotid artery stenting, 82% of whom had tandem lesions, treated with either aspirin or tirofiban bolus with infusion at lower doses that the aforementioned trial [13]. The primary efficacy outcome of in-stent thrombosis and the primary safety outcome of symptomatic ICH within the first 24 hours were significantly lower in the tirofiban group, without differences in functional outcomes at 90 days. The same investigators are currently enrolling patients in a randomized controlled trial coined ATILA [14]. Patients will be included who have tandem occlusions undergoing MT with stent placement and are either treated with IV aspirin or IV tirofiban, with the goal of measuring in-stent thrombosis and ICH rates. Results are expected to be published early next year and will be pivotal in guiding management of this subset of patients.

## Conclusions

This particular patient's case demonstrates the use of tirofiban as a perioperative antithrombotic to reduce the risk of in-stent thrombosis from carotid artery stenting for a tandem occlusion during MT. Identifying risk factors associated with increased ICH associated with tirofiban administration, such as preoperative IV fibrinolytic administration, initial NIHSS and ASPECTS score, time from symptom onset to recanalization, and tirofiban dosage, were important factors in the decision to carry out this treatment in our patient, as many characteristics did not require a one-size-fits-all approach. Although our patient did experience hemorrhagic conversion, the outcomes of our case were similar to other published studies showing no increased risk of symptomatic intracranial hemorrhage and a favorable long-term functional outcome.
